# Supplementary material for: Proline utilization A controls bacterial pathogenicity by sensing its substrate and cofactors
Source: Commun Biol. 2022 May 25;5:496. doi: 10.1038/s42003-022-03451-4 (PMC9132996; doi:10.1038/s42003-022-03451-4)
Supplement: Supplementary file 2 — Supplementary Information [file 42003_2022_3451_MOESM2_ESM.pdf]

Proline utilization A controls bacterial pathogenicity by sensing its substrate and cofactors

Peiyi Ye[1,2,3], Xia Li[1,3], Binbin Cui[1,3], Shihao Song[1,2,3], Fangfang Shen[2], Xiayu Chen[1], Gerun Wang[1], Xiaofan Zhou[2] and Yinyue Deng[1, *]

[1]*School of Pharmaceutical Sciences (Shenzhen), Shenzhen Campus of Sun Yat-sen University, Sun Yat-sen University, Shenzhen 518107, China*

[2]*Integrative Microbiology Research Center, College of Plant Protection, South China Agricultural University, Guangzhou 510642, China*

[3]The authors contributed equally

***Corresponding author:**

Yinyue Deng: dengyle@mail.sysu.edu.cn

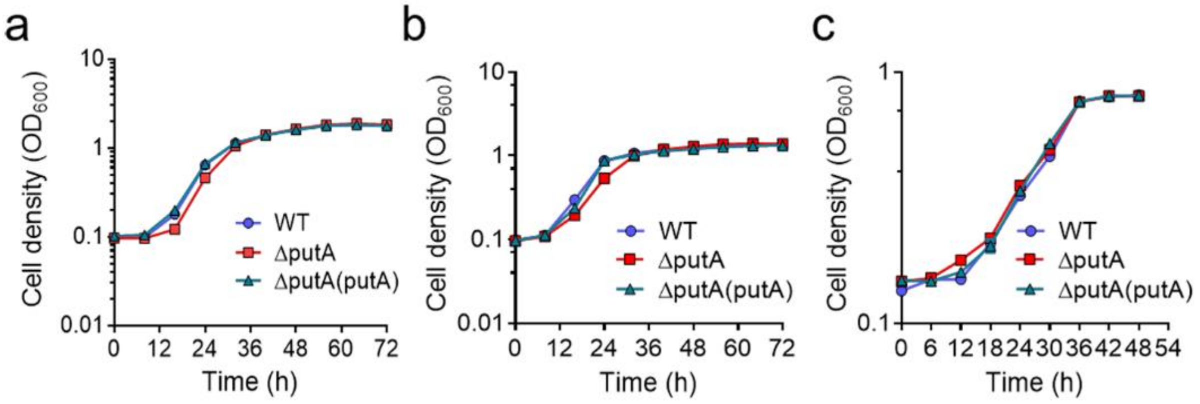

Supplementary Figure 1. Effect of *putA* on the growth of *R. solanacearum*. Cells were inoculated in triplicate at 28°C in a low-intensity shaking model using the Bioscreen-C automated growth curve analysis system. The experiments were started at an initial OD_{600} of 0.1 in TTC medium **(a)**, SP medium **(b)**, and MM medium **(c)**. Results in **a, b, c** are mean ± standard deviations of three independent experiments.

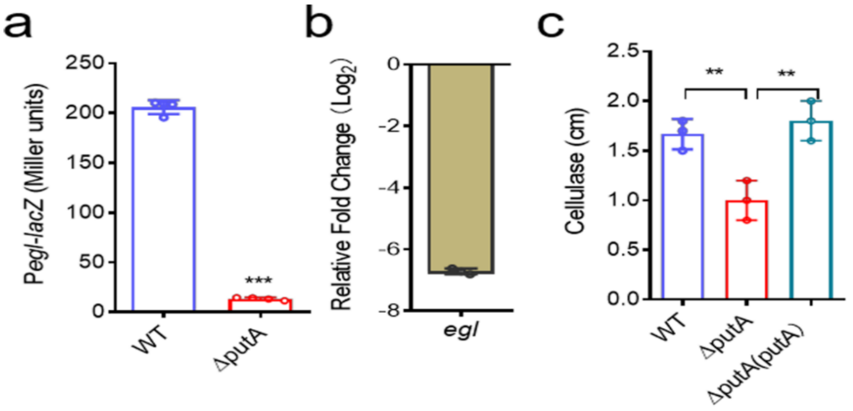

Supplementary Figure 2. Effect of *putA* on the cellulase production of *R. solanacearum*. a, Expression of *RSp0162* (*egl*) was evaluated by assessing the β-galactosidase activity of the *RSp0162*-*lacZ* transcriptional fusions in the GMI1000 wild-type and *putA* mutant strains. **b**, Expression levels of the gene encoding cellulase (*RSp0162*, *egl*) in the *putA* mutant strain compared to the wild-type strain GMI1000. **c**, The wild-type, *putA* mutant, and *putA* complement strains were tested for cellulase activity. Results in **a, b, c** are mean ± standard deviations of three independent experiments. *, $p < 0.05$; **$p < 0.01$; ***$p < 0.001$ (unpaired t test).

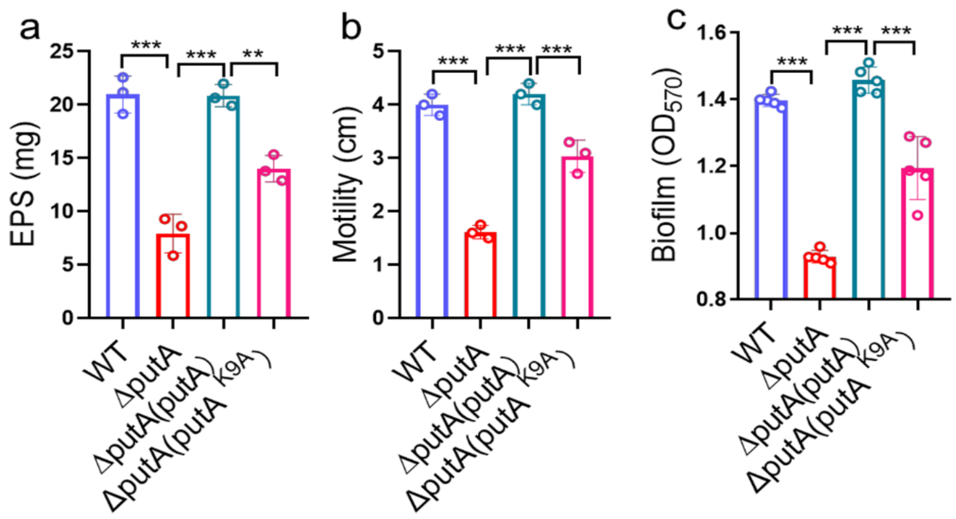

Supplementary Figure 3. Effects of PutA^{K9A} on virulence-related phenotypes. The wild-type strain, the *putA* mutant strain, the *putA* complemented strain and the *putA^{K9A}* complemented strain were evaluated for the following virulence-related phenotypes: EPS production (**a**), motility activity (**b**) and biofilm formation (**c**). Results in **a, b, c** are mean ± standard deviations of three or five independent experiments. *, $p < 0.05$; **$p < 0.01$; ***$p < 0.001$ (unpaired *t* test).

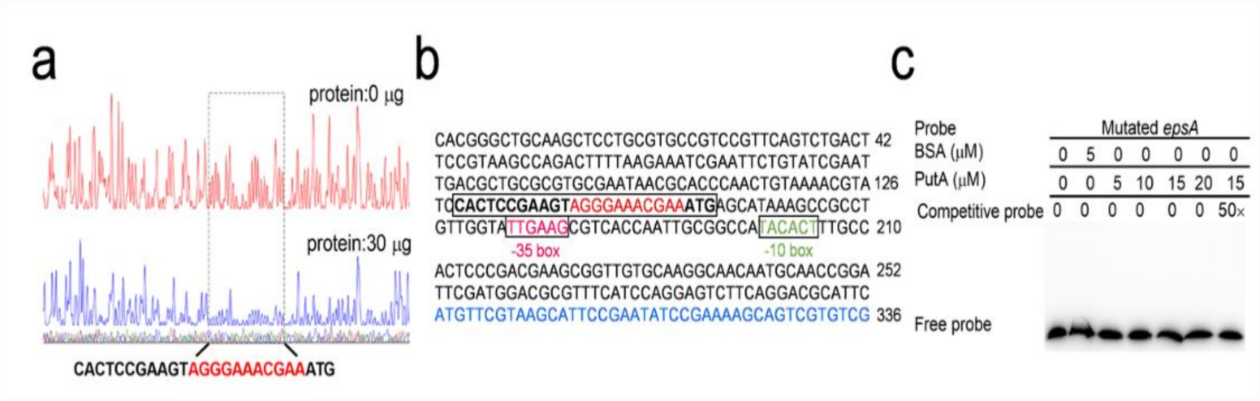

Supplementary Figure 4. Analysis of the PutA binding site in the promoter region of *epsA*. a, Identification of the DNA fragment in the *epsA* promoter protected by PutA from DNase I digestion. The PutA binding site in the promoter of *epsA* is shown in bold red letters. **b**, The structure of the *epsA* promoter. The PutA binding site (the red letters enclosed in the box) was determined to be located in the region upstream of the -35 position (pink) and -10 position (green). The transcription start site in the *epsA* gene is coloured blue. **c**, Analysis of the binding between PutA and the mutated *epsA* promoter with deletion of the PutA binding sequence. EMSA analysis was performed *in vitro*. A biotin-labelled 311-bp mutated *epsA* promoter DNA probe was used for the protein binding assay. No protein–DNA complexes formed when different concentrations of PutA protein were incubated with the probe at room temperature for 30 min. In **c** experiment was performed three times and representative images from one experiment are shown.

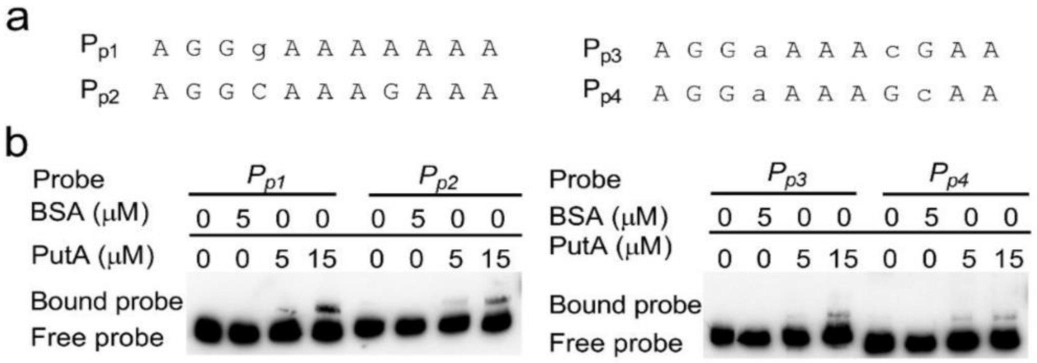

Supplementary Figure 5. Analysis of the binding of PutA to promoters with sequences similar to the binding site of PutA in the promoter of *epsA*. a, The similar sequences selected from *R. solanacearum*. **b**, EMSA analysis of the binding of PutA to the selected promoters *in vitro*. Biotin-labelled 157-bp P*p1*, 180-bp P*p2*, 184-bp P*p3* and 159-bp P*p4* promoter DNA probes were used for the protein binding assay. In **b** experiment was performed three times and representative images from one experiment are shown.

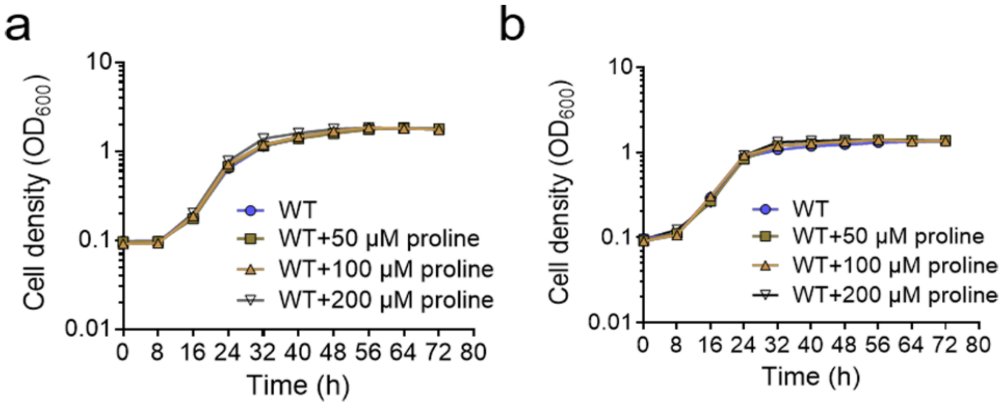

Supplementary Figure 6. Analysis of the growth of *R. solanacearum* cells with the addition of L-proline at different concentrations. Cells were inoculated in triplicate at 28°C in a low-intensity shaking model using the Bioscreen-C automated growth curve analysis system. The experiment was started at an initial OD_{600} of 0.1 in TTC medium **(a)** and SP medium **(b)**. Results in **a, b** are mean ± standard deviations of three independent experiments.

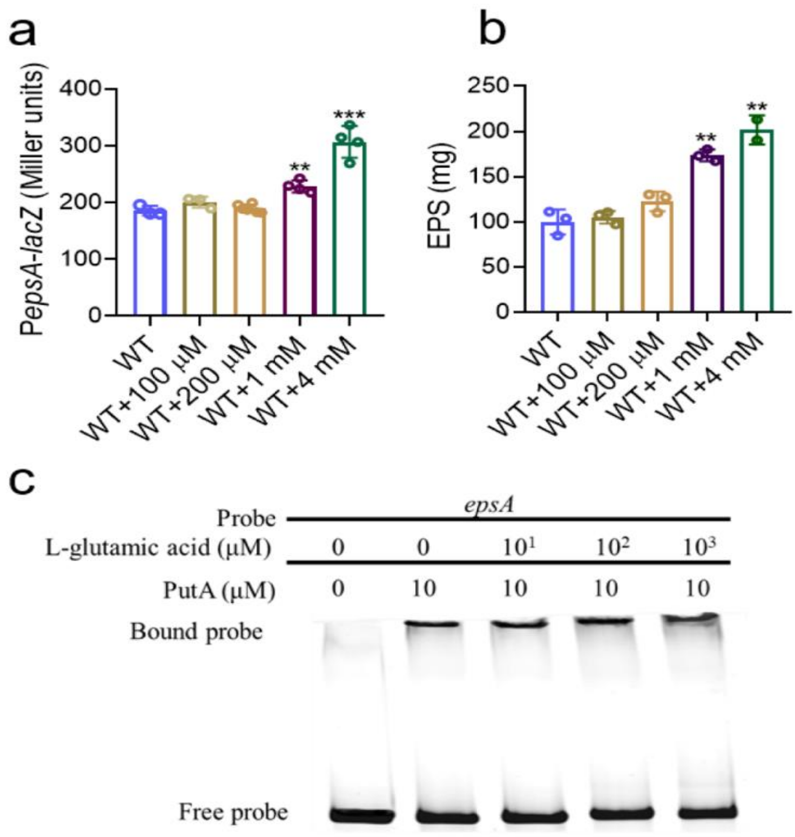

Supplementary Figure 7. Effects of L-glutamic acid on the regulatory activity of PutA in EPS biosynthesis. a, Expression of *epsA* was measured by assessing the *β*-galactosidase activity of *epsA*-*lacZ* transcriptional fusions in the wild-type with the addition of different concentrations of L-glutamic acid. **b**, Effects of L-glutamic acid on EPS production in the wild-type strain. **c**, EMSA analysis of the effect of L-glutamic acid on the binding of PutA to the *epsA* promoter *in vitro*, in which a biotin-labelled 336-bp *epsA* promoter DNA probe was used for the protein binding assay. Results in **a, b** are mean ± standard deviations of three independent experiments. *, $p < 0.05$; **$p < 0.01$; ***$p < 0.001$ (unpaired *t* test). In **c** experiment was performed three times and representative images from one experiment are shown.

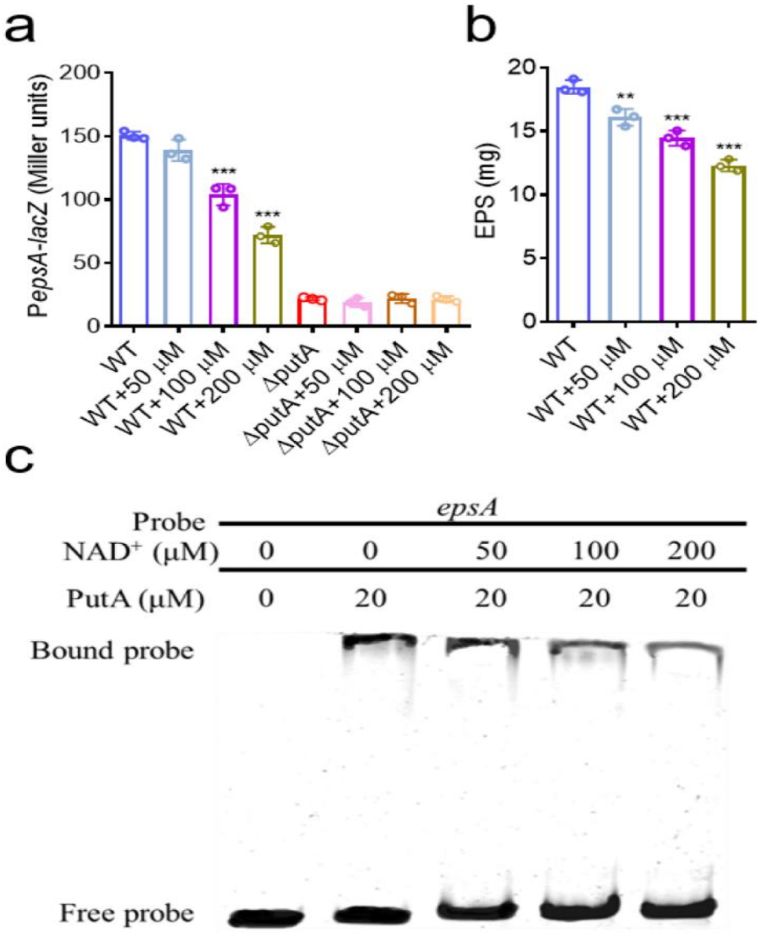

Supplementary Figure 8. Effects of NAD$^+$ on the regulatory activity of PutA in EPS biosynthesis. **a**, Expression of *epsA* was measured by assessing the *β*-galactosidase activity of *epsA*-*lacZ* transcriptional fusions in the wild-type and *putA* mutant strains with the addition of different concentrations of NAD$^+$. **b**, Effects of NAD$^+$ on EPS production in the wild-type strain. **c**, EMSA analysis of the effect of NAD$^+$ on the binding of PutA to the *epsA* promoter *in vitro*, in which a biotin-labelled 336-bp *epsA* promoter DNA probe was used for the protein binding assay. Results in **a, b** are mean ± standard deviations of three independent experiments. *, p < 0.05; **p < 0.01; ***p < 0.001 (unpaired t test). In **c** experiment was performed three times and representative images from one experiment are shown.

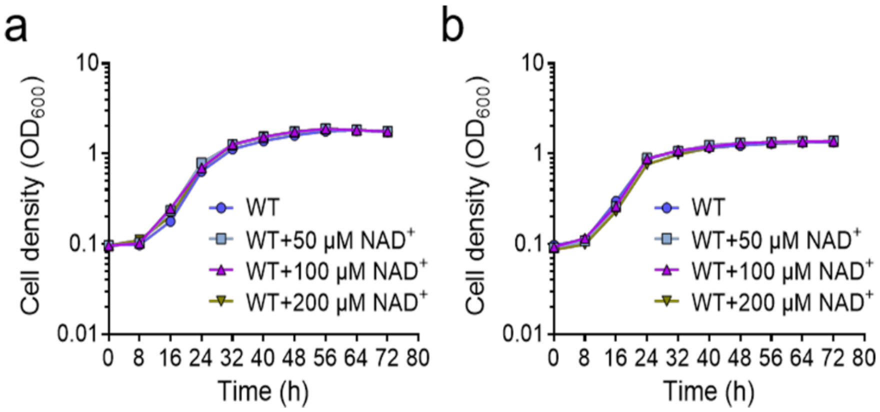

Supplementary Figure 9. Analysis of the growth of *R. solanacearum* cells with the addition of NAD^+ at different concentrations. Cells were inoculated in triplicate at 28°C in a low-intensity shaking model using the Bioscreen-C automated growth curve analysis system. The experiment was started at an initial OD_{600} of 0.1 in TTC medium **(a)** and SP medium **(b)**. Results in **a, b** are mean ± standard deviations of three independent experiments.

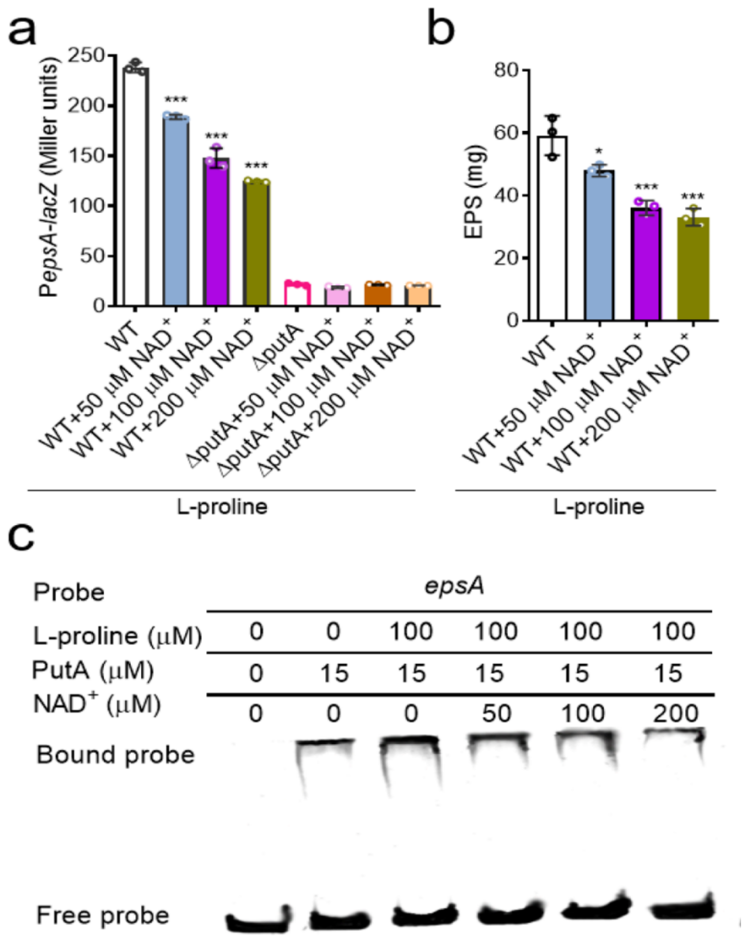

Supplementary Figure 10. Effects of NAD$^+$ on the regulatory activity of PutA in EPS biosynthesis in the presence of L-proline. a, Expression of *epsA* was evaluated by assessing the *β*-galactosidase activity of the *epsA*-*lacZ* transcriptional fusions in the wild-type and *putA* mutant strains with the addition of different concentrations of NAD$^+$ in the presence of 200 µM L-proline. **b**, Effect of NAD$^+$ on EPS production in the wild-type strain in the presence of 200 µM L-proline. **c**, EMSA analysis of the effect of NAD$^+$ on the binding of PutA to the *epsA* promoter in the presence of L-proline *in vitro*. A biotin-labelled 336-bp *epsA* promoter DNA probe was used for the protein binding assay. Results in **a, b** are mean ± standard deviations of three independent experiments. *, $p < 0.05$; **$p < 0.01$; ***$p < 0.001$ (unpaired *t* test). In **c** experiment was performed three times and representative images from one experiment are shown.

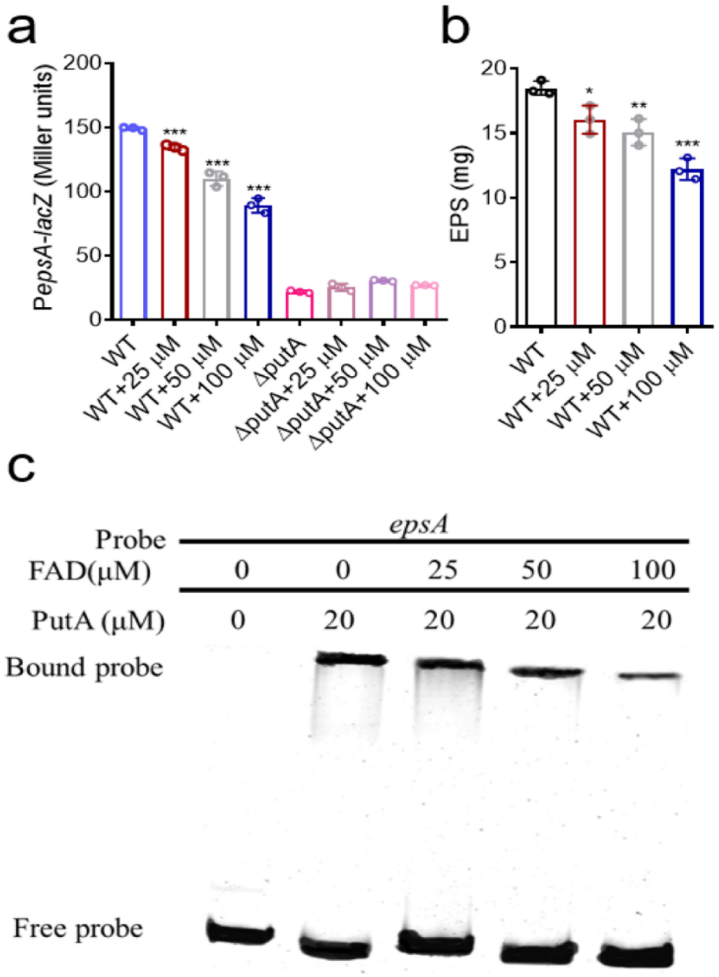

Supplementary Figure 11. Effects of FAD on the regulatory activity of PutA on EPS biosynthesis. a, Expression of *epsA* was evaluated by assessing the *β*-galactosidase activity of *epsA*-*lacZ* transcriptional fusions in the wild-type and *putA* mutant strains with the addition of different concentrations of FAD. **b**, Effect of FAD on EPS production in the wild-type strain. **c**, EMSA analysis of the effect of FAD on the binding of PutA to the *epsA* promoter *in vitro*. A biotin-labelled 336-bp *epsA* promoter DNA probe was used for the protein binding assay. Results in **a, b** are mean ± standard deviations of three independent experiments. *, $p < 0.05$; **$p < 0.01$; ***$p < 0.001$ (unpaired *t* test). In **c** experiment was performed four times and representative images from one experiment are shown.

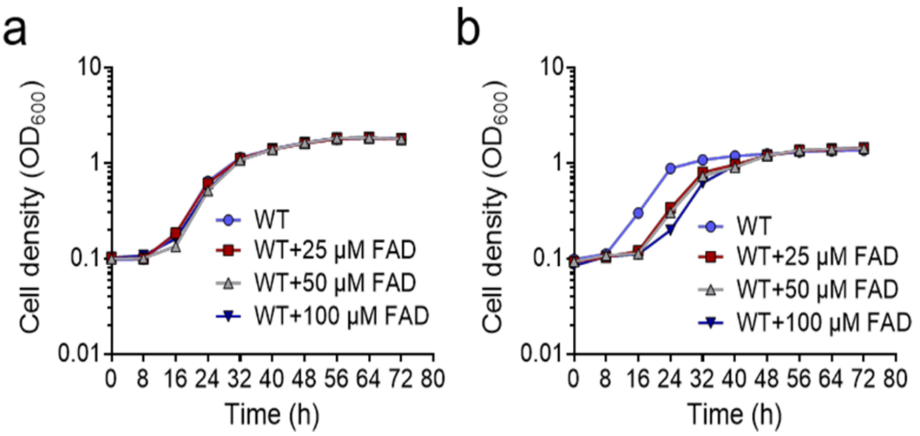

Supplementary Figure 12. Analysis of the growth of *R. solanacearum* cells with the addition of FAD at different concentrations. Cells were inoculated in triplicate at 28°C in a low-intensity shaking model using the Bioscreen-C automated growth curve analysis system. The experiment was started at an initial OD_{600} of 0.1 in TTC medium **(a)** and SP medium **(b)**. Results in **a, b** are mean ± standard deviations of three independent experiments.

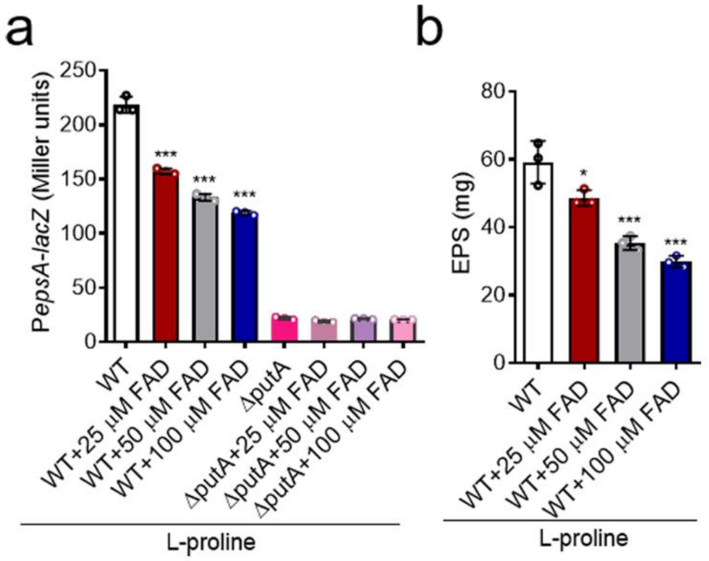

Supplementary Figure 13. Effects of FAD on the regulatory activity of PutA in EPS biosynthesis in the presence of L-proline. a, Expression of *epsA* was evaluated by assessing the *β*-galactosidase activity of the *epsA*-*lacZ* transcriptional fusions in the wild-type and *putA* mutant strains with addition of different concentrations of FAD in the presence of 200 μM L-proline. **b**, Effect of FAD on EPS production in the wild-type strain in the presence of 200 μM L-proline. Results in **a, b** are mean ± standard deviations of three independent experiments. *, $p < 0.05$; **$p < 0.01$; ***$p < 0.001$ (unpaired *t* test).

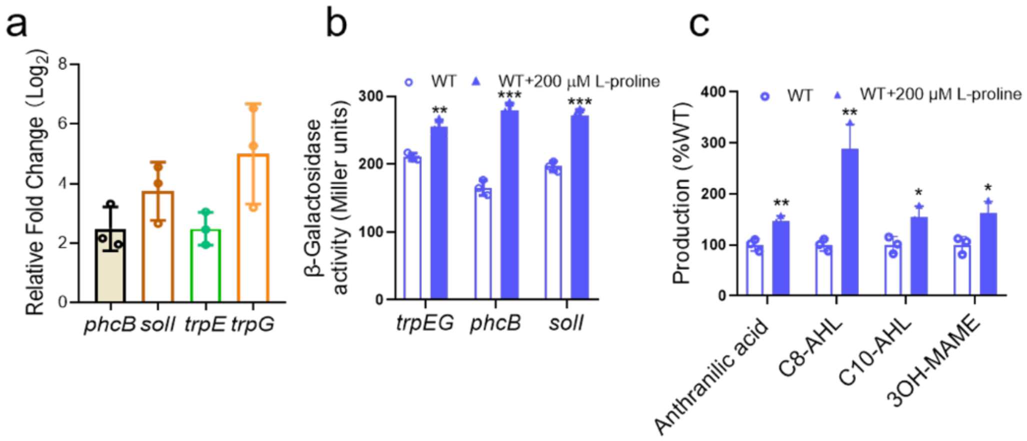

Supplementary Figure 14. Effects of PutA on the signalling systems in the presence of L-proline. a, The effects of *putA* on the expression of signal synthase-encoding genes were evaluated by RT-qPCR with addition of 200μM L-proline (OD_{600}=1.0). **b,** Expression of *trpEG*, *phcB* and *solI* by assessing the β-galactosidase activity of the *trpEG-lacZ*, *phcB-lacZ* and *solI-lacZ* transcriptional fusions in the wild-type strain in the presence or absence of 200μM L-proline (OD_{600}=3.0). **c,** Signal production in the wild-type strains in the presence or absence of 200μM L-proline was analysed by using UPLC-MS spectrometry. The amount of each signal in the *R. solanacearum* wild-type strain was arbitrarily defined as 100% and used to normalize the amount of that signal in the wild-type strain in the presence of L-proline **(c)**. Results in **a, b, c** are mean ± standard deviations of three or four independent experiments. *, $p < 0.05$; **$p < 0.01$; ***$p < 0.001$ (unpaired *t* test).

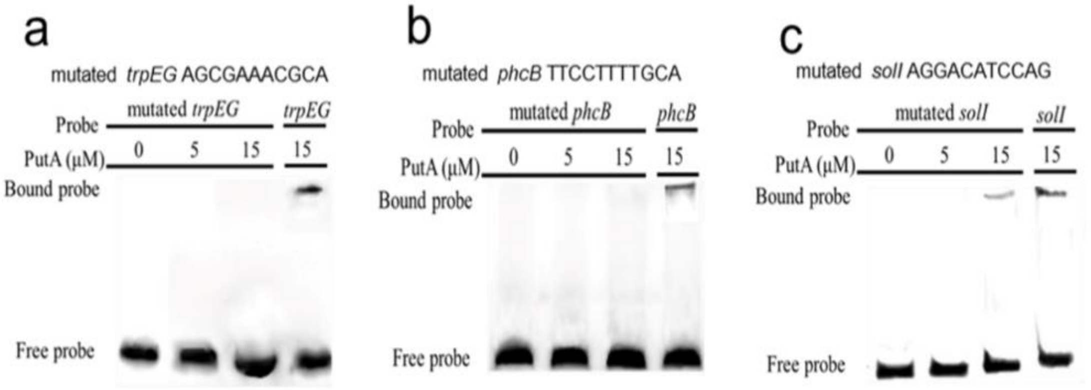

Supplementary Figure 15. Analysis of the binding of PutA to the mutated promoters with deletion of sequences similar to the binding site of PutA in the promoter of *epsA*. a, A biotin-labelled 233-bp mutated *trpEG* promoter DNA probe with AGCGAAACGCA deleted was used for the protein binding assay. **b,** A biotin-labelled 316-bp mutated *phcB* promoter DNA probe with TTCCTTTTGCA deleted was used for the protein binding assay. **c,** A biotin-labelled 300-bp mutated *solI* promoter DNA probe with AGGACATCCAG deleted was used for the protein binding assay. Each experiment was performed three times and representative images from one experiment are shown.

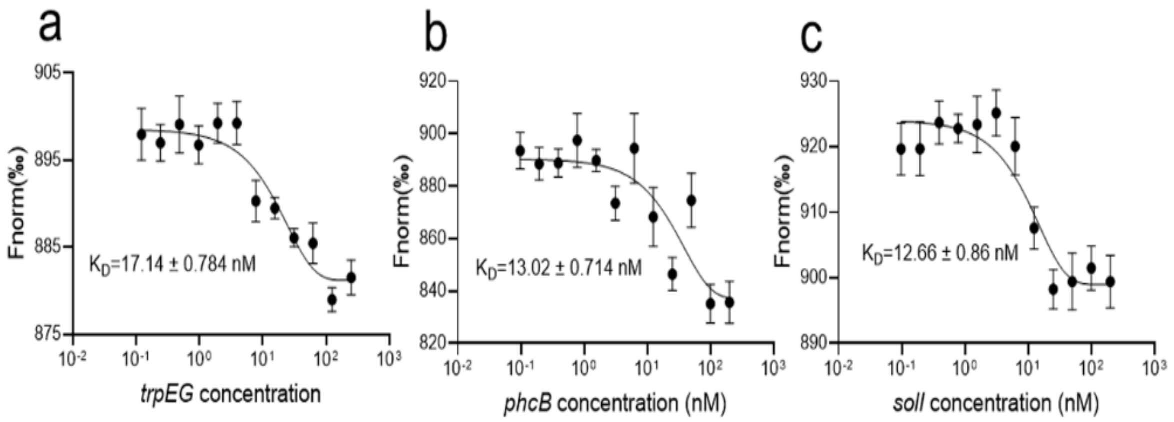

Supplementary Figure 16. MST analysis of the interaction of promoter DNA and PutA. MST analysis of the PutA binding to *trpEG* **(a)**, *phcB* **(b)** and *solI* **(c)** promoter DNA probe. “Fnorm (‰)” indicates the fluorescence time trace changes in MST response. Results in **a, b, c** are mean ± standard deviations of three independent experiments.

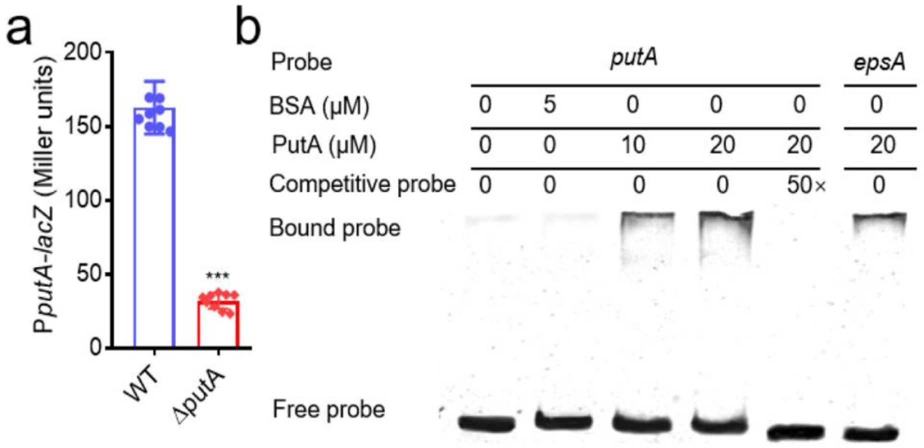

Supplementary Figure 17. Effect of PutA on the expression of *putA*. a, The expression of *putA* was evaluated by assessing the *β*-galactosidase activity of the *putA*-*lacZ* fusion transcripts in the wild-type and *putA* mutant strains. **b**, EMSA analysis of the binding of PutA to the *putA* promoter *in vitro*. A biotin-labelled 315-bp *putA* promoter DNA probe was used for the protein binding assay. Results in **a** is means ± standard deviations of three independent experiments. **$p < 0.01$; ***$p < 0.001$ (unpaired *t* test). In **b** experiment was performed three times and representative images from one experiment are shown.

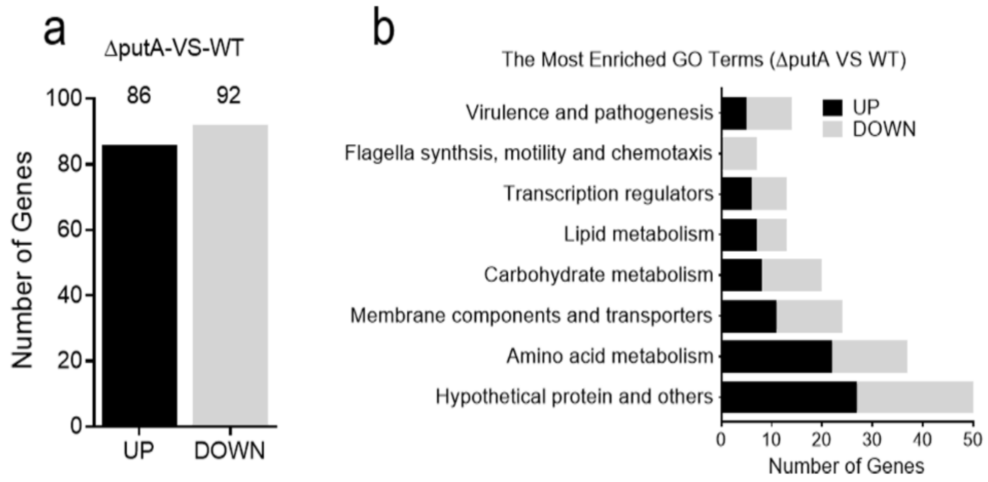

Supplementary Figure 18. Differential gene expression profiles between the *R. solanacearum* GMI1000 *putA* mutant strain and the wild-type strain as measured by RNA-Seq (Log_2 fold-change$\geqslant$1.0). a, The number of genes were upregulated (UP) and downregulated (DOWN) in the *putA* mutant strain compared with the wild-type strain. **b,** GO term enrichment analysis of differentially expressed genes between the *putA* mutant strain and the wild-type strain.

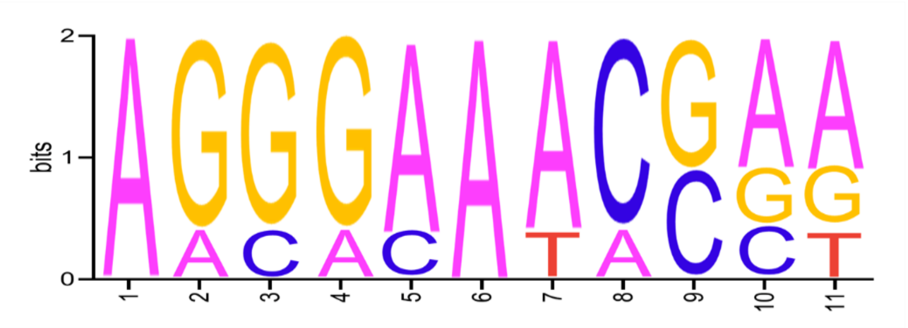

Supplementary Figure 19. Sequence analysis of PutA binding sites in the promoters of the signal synthase-encoding genes.

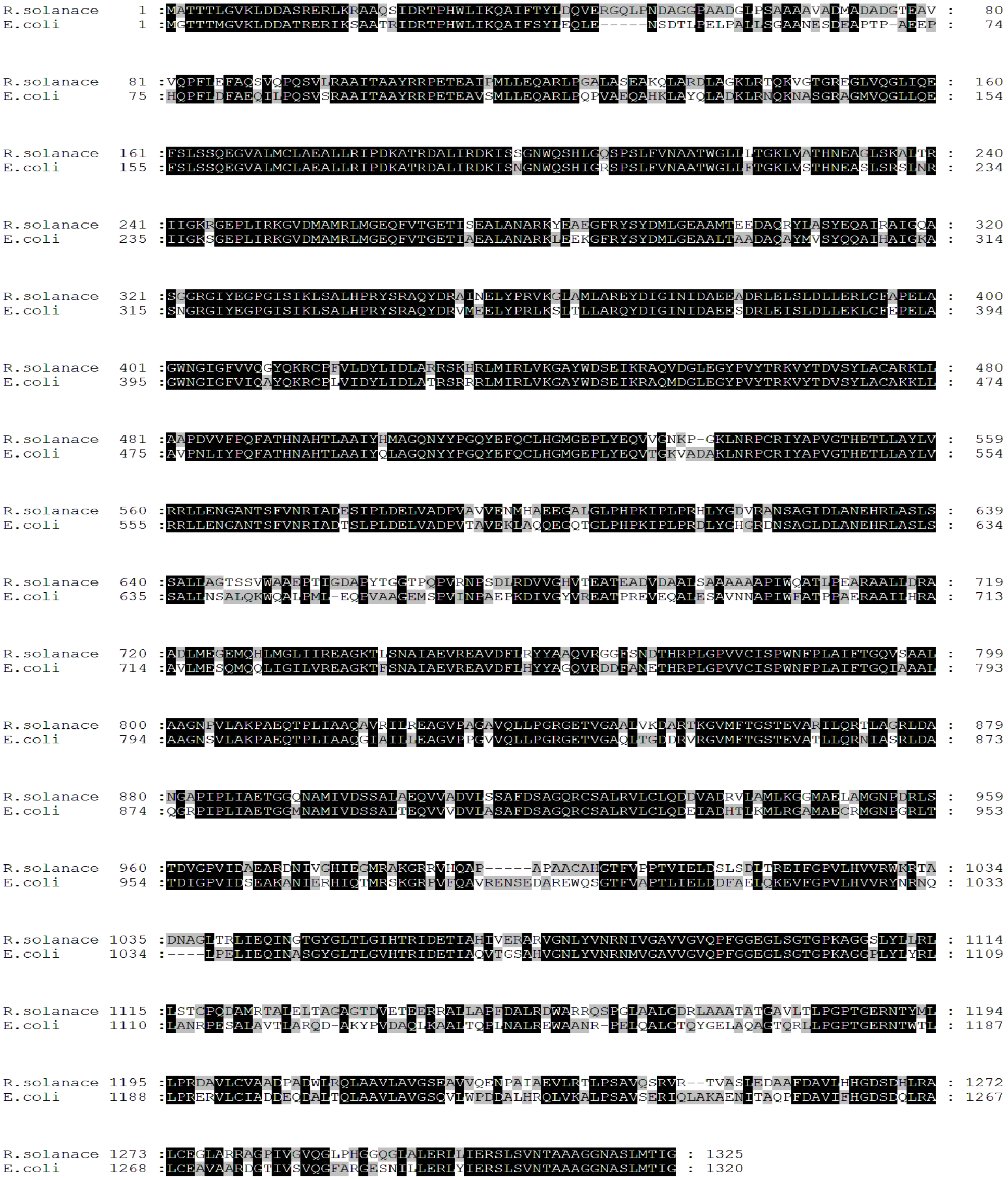

Supplementary Figure 20. Alignment of protein sequences of PutA with the homolog from *E. coli*. The black and gray shading indicates the identical and similar residues, respectively.

Supplementary Table 1 Location of transposon insertions identified in *R. solanacearum* GMI1000

Inserted gene	Number of different insertion mutants	Gene product
RS_RS03495	2	Channel protein TolC
RS_RS03280	1	CpaF family protein
trpC	2	Indole-3-glycerol phosphate synthase 1
RS_RS22015	4	EPS Ⅰ polysaccharide export inner membrane protein EpsF
RS_RS13395	3	Type 4 fimbrial biogenesis PilY1 signal peptide
putA	1	Trifunctional transcriptional regulator / proline dehydrogenase / L-glutamate gamma-semialdehyde dehydrogenase
RS_RS05670	1	Ferredoxin-NADP(+) reductase
RS_RS19430	1	Hypothetical protein
RS_RS14145	1	Dephospho-CoA kinase

Supplementary Table 2 List of genes differentially expressed in the *putA* mutant compared to the wild-type strain (Log_2 fold change ⩾ 1). Significantly differentially expressed genes were determined by Cufflinks after Benjamini-Hochberg correction. The fold change is the ratio of the mutant FPKM to the wild-type FPKM.

Class	Gene ID	Fold change	Description
Flagella synthesis, motility and chemotaxis			
	RS_RS23925	-3.31	chemotaxis protein
	RS_RS18800	-1.98	flagellar basal body rod protein FlgC
	RS_RS19040	-1.75	flagellar motor switch protein FliG
	RS_RS19045	-1.51	flagellar assembly protein FliH
	RS_RS18805	-1.48	flagellar basal body rod modification protein FlgD
	RS_RS18980	-1.27	flagellar basal body-associated protein FliL
	RS_RS23935	-1.10	flagellar motor protein MotA
Membrane components and transporters			
	RS_RS22035	-6.17	tyrosine protein kinase
	RS_RS23240	-6.14	membrane protein
	RS_RS24485	-4.87	membrane protein
	RS_RS06720	-4.11	aliphatic sulfonate ABC transporter permease SsuC
	RS_RS19655	-2.53	signal transduction ggdef domain protein
	RS_RS17695	-2.04	membrane protein
	RS_RS08775	-2.02	porin
	RS_RS12255	-1.96	branched chain amino acid ABC transporter substrate-binding protein
	RS_RS12235	-1.73	ABC transporter ATP-binding protein
	RS_RS12240	-1.58	ABC transporter ATP-binding protein
	RS_RS24945	-1.56	D-xylose ABC transporter substrate-binding protein XylF
	RS_RS02795	-1.33	type 4 fimbrial pilin signal peptide protein
	RS_RS07670	-1.23	phosphate ABC transporter substrate-binding protein PstS
	RS_RS21805	1.38	NarK/NasA family nitrate transporter
	RS_RS21195	1.99	iron dicitrate transporter FecR
	RS_RS23735	2.04	ABC transporter ATP-binding protein
	RS_RS23795	2.06	sulfonate ABC transporter substrate-binding protein
	RS_RS23740	2.14	ABC transporter permease
	RS_RS23910	2.45	membrane protein
	RS_RS22085	2.58	membrane protein
	RS_RS04025	2.68	membrane protein
	RS_RS23310	3.32	membrane protein
	RS_RS01070	3.89	MFS transporter
	RS_RS23800	4.20	membrane protein
Transcription regulators			
	RS_RS24060	-3.96	methionine--tRNA ligase
	RS_RS16470	-3.35	transcriptional activator protein SolR
	RS_RS20830	-3.01	autoinducer binding domain-containing protein
	RS_RS14805	-2.83	BolA family transcriptional regulator
	RS_RS13750	-2.11	LysR family transcriptional regulator PhcA
	RS_RS04735	-1.76	RNA helicase
	RS_RS23940	-1.52	transcriptional activator FlhC
	RS_RS00325	2.35	LysR family transcriptional regulator
	RS_RS23725	2.39	regulatory protein NosR
	RS_RS17670	3.21	TetR/AcrR family transcriptional regulator
	RS_RS00800	4.29	TetR/AcrR family transcriptional regulator
	RS_RS06490	5.03	MarR family transcriptional regulator
	RS_RS10620	5.75	GntR family transcriptional regulator
Carbohydrate metabolism			
	RS_RS22030	-6.36	UDP-N-acetyl glucosamine 2-epimerase
	RS_RS21965	-6.16	dTDP-glucose 2C6-dehydratase
	RS_RS20225	-4.96	3-hydroxyisobutyrate dehydrogenase MmsB
	RS_RS19230	-4.45	alcohol dehydrogenase catalytic domain-containing protein
	RS_RS22535	-4.04	MBL fold metallo-hydrolase
	RS_RS22540	-3.82	SDR family NAD(P)-dependent oxidoreductase
	RS_RS21875	-3.80	4-hydroxybutyrate dehydrogenase
	RS_RS23965	-3.45	hydrolase
	RS_RS06725	-3.36	ATP-binding cassette domain-containing protein
	RS_RS17735	-2.57	2-methylcitrate synthase
	RS_RS24610	-1.75	6-phosphogluconolactonase
	RS_RS13685	-1.52	SAM-dependent methyltransferase PhcB

	RS_RS12980	1.32	limonene-1,2-epoxide hydrolase
	RS_RS00960	1.87	alcohol dehydrogenase
	RS_RS22465	2.07	alpha/beta hydrolase
	RS_RS19050	2.26	ATP synthase
	RS_RS21760	2.37	anaerobic ribonucleoside triphosphate reductase
	RS_RS21505	2.52	Gfo/Idh/MocA family oxidoreductase
	RS_RS19100	3.05	fatty acyl-AMP ligase
	RS_RS22480	4.38	alpha/beta hydrolase
Amino acid metabolism			
	RS_RS22040	-7.48	protein-tyrosine-phosphatase
	RS_RS20360	-3.51	5-methyltetrahydropteroyltriglutamate--homocysteine methyltransferase
	RS_RS13250	-3.13	histidine ammonia-lyase HutH
	RS_RS23970	-2.80	peptide synthetase
	RS_RS13255	-2.72	urocanate hydratase
	RS_RS13240	-2.71	imidazolonepropionase
	RS_RS14430	-2.66	anthranilate synthase component I
	RS_RS14435	-2.58	aminodeoxychorismate/anthranilate synthase component II
	RS_RS19720	-2.55	peptidase M1
	RS_RS24725	-2.47	catalase
	RS_RS20850	-2.31	aminoacyl-tRNA synthet
	RS_RS13245	-2.17	formimidoylglutamase
	RS_RS20845	-1.55	cystathionine gamma-synthase
	RS_RS17975	-1.24	NIFS-like protein
	RS_RS00560	-1.13	2-nitropropane dioxygenase
	RS_RS06410	1.10	probable cytochrome c oxidase (subunit III) transmembrane protein
	RS_RS06745	1.22	sulfate ABC transporter permease subunit CysT
	RS_RS06405	1.23	cytochrome oxidase
	RS_RS10145	1.26	phenylacetic acid degradation protein
	RS_RS20010	1.52	phenylacetate-CoA oxygenase subunit PaaB
	RS_RS00780	1.61	arginase
	RS_RS20005	1.63	phenylacetate-CoA oxygenase subunit PaaA
	RS_RS20025	1.64	phenylacetic acid degradation protein
	RS_RS19480	1.67	ornithine cyclodeaminase
	RS_RS06395	1.85	cytochrome c oxidase, cbb3-type subunit I
	RS_RS10110	1.85	glutathione S-transferase
	RS_RS21820	2.00	nitrate reductase 2 (NRZ), beta subunit
	RS_RS18665	2.22	2-nitropropane dioxygenase
	RS_RS06400	2.31	peptidase S41
	RS_RS24370	2.45	nitric-oxide reductase large subunit
	RS_RS00145	2.49	4-aminobutyrate transaminase GabT
	RS_RS23720	2.50	TAT-dependent nitrous-oxide reductase
	RS_RS07545	3.42	tyrosinase
	RS_RS21815	3.54	nitrate reductase subunit alpha
	RS_RS21810	3.77	Nitrate/nitrite transporter (plasmid)
	RS_RS20450	4.58	tryptophan 2,3-dioxygenase
	RS_RS24360	5.31	probable major anaerobically induced outer membrane transmembrane protein
Lipid metabolism			
	RS_RS20835	-4.12	acyl-homoserine-lactone synthase
	RS_RS20215	-3.93	enoyl-CoA hydratase/isomerase family protein
	RS_RS20220	-3.81	enoyl-CoA hydratase
	RS_RS16465	-3.61	acyl-homoserine-lactone synthase SolI
	RS_RS20870	-2.41	beta-ketoacyl-ACP synthase
	RS_RS18695	-1.72	2-isopropylmalate synthase 2
	RS_RS08885	1.32	3-hydroxyacyl-CoA dehydrogenase
	RS_RS15725	1.86	zinc-dependent alcohol dehydrogenase
	RS_RS16455	2.36	coproporphyrinogen III oxidase
	RS_RS15300	2.55	glycerol-3-phosphate dehydrogenase
	RS_RS24625	2.77	universal stress protein UspA
	RS_RS15335	3.58	glycerol kinase
	RS_RS01000	3.71	phosphoribosylpyrophosphate synthetase
Virulence and pathogenesis			
	RS_RS17915	-4.73	endoglucanase precursor (endo-1,4-beta-glucanase)(cellulase) protein EGL
	RS_RS22615	-3.51	type VI secretion protein

	RS_RS22045	-3.44	EPS I polysaccharide export outer membrane protein EpsA
	RS_RS22035	-3.05	polysaccharide biosynthesis tyrosine autokinase EpsB
	RS_RS20770	-2.54	type VI secretion protein VasK
	RS_RS22020	-1.98	EPS I polysaccharide export inner membrane protein EpsE
	RS_RS22030	-1.54	udp-n-acetylglucosamine 2-epimerase (udp-glcnac-2-epimerase) protein EpsC
	RS_RS26155	-1.42	probable exoglucanase a (1,4-beta-cellobiosidase) protein
	RS_RS22025	-1.23	ndp-n-acetyl-d-galactosaminuronic acid dehydrogenase . oxidoreductase protein EpsD
	RS_RS01205	1.01	type III secretion system effector protein
	RS_RS06765	1.36	type III effector protein
	RS_RS21295	2.89	EscN/YscN/HrcN family type III secretion system ATPase
	RS_RS21305	4.19	EscT/YscT/HrcT family type III secretion system export apparatus protein
	RS_RS04330	4.75	YOPP/AvrRxv family protein
Hypothetical protein and others			
	RS_RS21270	-6.83	hypothetical protein
	RS_RS19745	-6.06	hypothetical protein
	RS_RS24290	-4.97	hypothetical protein
	RS_RS24045	-4.39	hypothetical protein
	RS_RS24285	-4.24	hypothetical protein
	RS_RS17970	-4.01	hypothetical protein
	RS_RS19245	-3.98	hypothetical protein
	RS_RS22605	-3.35	hypothetical protein
	RS_RS19240	-3.32	cupin domain-containing protein
	RS_RS22610	-3.31	hypothetical protein
	RS_RS10175	-3.27	hypothetical protein
	RS_RS27285	-3.21	hypothetical protein
	RS_RS19080	-3.18	hypothetical protein
	RS_RS07455	-3.04	DUF1653 domain-containing protein
	RS_RS01140	-2.96	hypothetical protein
	RS_RS26530	-2.66	hypothetical protein
	RS_RS18100	-2.60	disulfide bond formation protein B
	RS_RS20355	-2.43	hypothetical protein
	RS_RS20525	-2.04	cytochrome P450
	RS_RS21360	-1.51	hypothetical protein
	RS_RS22805	-1.40	hypothetical protein
	RS_RS14440	-1.13	DUF1311 domain-containing protein
	RS_RS25215	-1.04	hypothetical protein
	RS_RS01575	1.12	hypothetical protein
	RS_RS26175	1.25	hypothetical protein
	RS_RS11245	1.36	hypothetical protein
	RS_RS01595	1.42	hypothetical protein
	RS_RS24190	1.67	hypothetical protein
	RS_RS25740	1.69	hypothetical protein
	RS_RS00900	1.72	DUF2892 domain-containing protein
	RS_RS10855	1.85	DUF2383 domain-containing protein
	RS_RS06325	1.96	hypothetical protein
	RS_RS04805	2.06	hypothetical protein
	RS_RS24635	2.19	hypothetical protein
	RS_RS10955	2.22	DUF1232 domain-containing protein
	RS_RS19095	2.31	hypothetical protein
	RS_RS06425	2.41	hypothetical protein
	RS_RS19220	2.46	hypothetical protein
	RS_RS16930	2.52	hypothetical protein
	RS_RS19020	2.65	hypothetical protein
	RS_RS15945	2.88	hypothetical protein
	RS_RS15440	3.06	hypothetical protein
	RS_RS07420	3.09	hypothetical protein
	RS_RS07550	3.25	hypothetical protein
	RS_RS07555	3.98	hypothetical protein
	RS_RS21185	4.42	hypothetical protein
	RS_RS21340	4.63	hypothetical protein
	RS_RS16485	5.13	hypothetical protein
	RS_RS19485	5.40	hypothetical protein
	RS_RS21230	5.99	hypothetical protein

375

Supplementary Table 3 Analysis of the homologues of *putA* in various bacterial

Bacteria	*putA* homologue Identity (%)	*putA* homologue Accession No.
Ralstonia		
R. solanacearum GMI1000	100	WP_071893022.1
R. pseudosolanacearum	99.62	WP_211906464.1
R. syzygii	94.88	WP_211904254.1
R. mannitolilytica	94.49	WP_104610819.1
R. pickettii	94.11	WP_004628663.1
R. insidiosa	92.65	WP_104656765.1
Pseudomonas		
P. capsici	72.60	WP_206401554.1
P. vranovensis	72.52	WP_028945626.1
P. aestus	72.50	WP_022640308.1
P. massiliensis	72.50	WP_040259610.1
P. putida	72.50	WP_064301470.1
P. frederiksbergensis	72.47	WP_071552264.1
P. batumici	72.46	WP_040063760.1
P. brassicacearum	72.46	WP_123423857.1
P. hunanensis	72.45	WP_103517985.1
P. fuscovaginae	72.43	WP_010453746.1
P. graminis	72.43	WP_172610475.1
P. lundensis	72.43	WP_083332949.1
P. piscis	72.43	WP_152899078.1
P. abietaniphila	72.37	WP_062379176.1
P. asplenii	72.37	WP_090202778.1
P. gingeri	72.37	WP_177072828.1
P. guariconensis	72.36	WP_196145100.1
P. monteilii	72.36	WP_196166374.1
P. alkylphenolica	72.35	WP_128321595.1
P. reidholzensis	72.35	WP_119146398.1
P. saponiphila	72.35	WP_092318977.1
P. wadenswilerensis	72.35	WP_115089182.1
P. indica	72.33	MBU3056109.1
P. asiatica	72.32	WP_182328877.1
P. asturiensis	72.30	WP_073168123.1
P. chlororaphis	72.30	WP_123573330.1
P. kilonensis	72.30	WP_024617974.1
P. laurentiana	72.30	WP_189395471.1
P. mandelii	72.30	WP_169857554.1
P. kielensis	72.28	WP_185817851.1
P. protegens	72.28	WP_047335503.1
P. saxonica	72.28	WP_146424707.1

P. weihenstephanensis	72.28	WP_203303079.1
P. parafulva	72.26	WP_039582802.1
P. foliumensis	72.22	WP_187521594.1
P. savastanoi	72.22	WP_122394875.1
P. thivervalensis	72.22	WP_053181383.1
P. donghuensis	72.20	WP_100782886.1
P. migulae	72.20	WP_182343216.1
P. cichorii	72.19	WP_201001533.1
P. canadensis	72.15	WP_028615457.1
P. prosekii	72.15	WP_121734301.1
P. entomophila	72.13	WP_181102701.1
P. allokribbensis	72.07	WP_192559596.1
P. azotoformans	72.07	WP_084301303.1
P. eucalypticola	72.07	WP_176569699.1
P. kribbensis	72.07	WP_134825242.1
P. reactans	72.07	WP_177001637.1
P. simiae	72.07	WP_045791375.1
P. caspiana	72.05	WP_087271265.1
P. helleri	72.05	WP_153381713.1
P. japonica	72.05	WP_042120370.1
P. cedrina	72.00	WP_076951789.1
P. floridensis	72.00	WP_083185594.1
P. gozinkensis	72.00	WP_192563245.1
P. reinekei	72.00	WP_075947280.1
P. yamanorum	72.00	WP_177041176.1
P. syringae	71.99	WP_047576415.1
P. fragi	71.98	WP_169869406.1
P. plecoglossicida	71.98	WP_181107288.1
P. psychrophila	71.98	WP_019411488.1
P. triticumensis	71.96	WP_187519742.1
P. lurida	71.94	WP_034105832.1
P. allii	71.92	WP_179029872.1
P. cremoris	71.92	WP_185704967.1
P. extremorientalis	71.92	WP_071492331.1
P. mucoides	71.92	WP_194940869.1
P. nabeulensis	71.92	WP_135308025.1
P. poae	71.92	WP_105695098.1
P. glycinae	71.90	WP_197869271.1
P. tolaasii	71.90	WP_177006706.1
P. viridiflava	71.90	WP_122485523.1
P. orientalis	71.89	WP_104501396.1
P. asuensis	71.88	WP_188866678.1
P. arsenicoxydans	71.85	WP_140668236.1

P. haemolytica	71.85	WP_153838708.1
P. kitaguniensis	71.85	WP_152748713.1
P. koreensis	71.85	WP_210709985.1
P. marginalis	71.85	WP_064054025.1
P. stutzeri	71.85	WP_204930763.1
P. trivialis	71.85	WP_049710366.1
P. umsongensis	71.85	WP_179054820.1
P. vancouverensis	71.85	WP_093227347.1
P. bohemica	71.83	WP_110950951.1
P. corrugata	71.83	WP_055135644.1
P. extremaustralis	71.83	WP_010563252.1
P. huaxiensis	71.83	WP_110971422.1
P. sichuanensis	71.83	WP_110995800.1
P. veronii	71.83	WP_198718329.1
P. carnis	71.77	WP_127880476.1
P. jessenii	71.77	WP_139053535.1
P. rhodesiae	71.77	WP_123479577.1
P. endophytica	71.75	WP_055104861.1
P. fildesensis	71.75	WP_048727730.1
P. synxantha	71.75	WP_078820115.1
P. antarctica	71.70	WP_064450346.1
P. granadensis	71.70	WP_090280545.1
P. iridis	71.70	WP_210702951.1
P. lactis	71.70	WP_169913010.1
P. libanensis	71.70	WP_057010685.1
P. lini	71.70	WP_050681741.1
P. mohnii	71.70	WP_090465537.1
P. salomonii	71.70	WP_177023469.1
P. sivasensis	71.70	WP_181643768.1
P. capeferrum	71.68	WP_181130721.1
P. mediterranea	71.68	WP_201868604.1
P. mosselii	71.68	WP_138219194.1
P. paralactis	71.68	WP_186559576.1
P. viciae	71.68	WP_135843363.1
P. lutea	71.65	WP_197872531.1
P. atacamensis	71.62	WP_136492375.1
P. gessardii	71.62	WP_076961761.1
P. grimontii	71.62	WP_090400927.1
P. kairouanensis	71.62	WP_135291937.1
P. laurylsulfatiphila	71.62	WP_104451077.1
P. laurylsulfativorans	71.62	WP_103397319.1
P. moorei	71.62	WP_090328349.1
P. panacis	71.62	WP_154841358.1

P. pisciculturae	71.60	WP_194937418.1
P. proteolytica	71.60	WP_169909194.1
P. costantinii	71.57	WP_177011609.1
P. matsuisoli	71.55	WP_188983317.1
P. neuropathica	71.55	WP_194935851.1
P. palleroniana	71.55	WP_090367233.1
P. helmanticensis	71.51	WP_134175893.1
P. luteola	71.50	WP_125889424.1
P. anatoliensis	71.47	WP_210701190.1
P. edaphica	71.47	WP_177034877.1
P. baetica	71.40	WP_100846477.1
P. fulva	71.40	WP_167338974.1
P. sagittaria	71.37	WP_092433980.1
Amantichitinum		
A. ursilacus	77.71	WP_053939191.1
Betaproteobacteria		
B. bacterium	92.27	NOZ15061.1
Cupriavidus		
C. necator	79.94	WP_153946340.1
C. metallidurans	79.59	WP_035884406.1
C. pauculus	79.37	WP_101679621.1
C. basilensis	79.19	WP_043350742.1
C. plantarum	78.75	WP_109585338.1
C. yeoncheonensis	78.71	WP_211946096.1
C. numazuensis	78.64	WP_211952752.1
C. pinatubonensis	78.56	WP_140950001.1
C. cauae	78.52	WP_150082051.1
C. gilardii	78.51	WP_208580388.1
C. campinensis	78.5	WP_144203378.1
C. alkaliphilus	77.94	WP_092316757.1
C. neocaledonicus	77.79	WP_018006193.1
C. oxalaticus	77.67	WP_063240874.1
C. taiwanensis	77.60	WP_116292522.1
C. lacunae	77.59	WP_115215780.1
Escherichia		
E. coli	72.30	MRF42240.1
Priestia		
P. aryabhattai	72.50	QPN45981.1
Pseudomonadales		
P. bacterium	71.77	MBH1968881.1
Silvimonas		
S. terrae	77.61	WP_184096792.1
S. amylolytica	77.24	WP_188688929.1

S. iriomotensis	76.78	WP_188701611.1

Supplementary Table 4 Bacterial strains and plasmids used in this study

**Strain or plasmid**	**Phenotype and/or characteristic(s)**[a]	**Source or reference**
R. solanacearum		
GMI1000	Wild-type strain of *R. solanacearum*	ATCCBAA-1114
GMI1000(P*epsA*-*lacZ*)	GMI1000 harboring the reporter construct P*epsA*-*lacZ*	1
ΔputA	Deletion mutant with *putA* being deleted	This study
ΔputA(putA)	Mutant ΔputA harboring the expression construct pLAFR3-*putA*	This study
ΔputA(putA ^{K9A})	Mutant ΔputA harboring the expression construct pLAFR3-*putA* ^{K9A}	This study
ΔputA(P*epsA*-*lacZ*)	Mutant ΔputA harboring the reporter construct P*epsA*-*lacZ*	This study
GMI1000(P*phcB*-*lacZ*)	GMI1000 harboring the reporter construct P*phcB*-*lacZ*	This study
ΔputA(P*phcB*-*lacZ*)	Mutant ΔputA harboring the reporter construct P*phcB*-*lacZ*	This study
GMI1000(P*solI*-*lacZ*)	GMI1000 harboring the reporter construct P*solI*-*lacZ*	This study
ΔputA(P*solI*-*lacZ*)	Mutant ΔputA harboring the reporter construct P*solI*-*lacZ*	This study
GMI1000(P*trpEG*-*lacZ*)	GMI1000 harboring the reporter construct P*trpEG*-*lacZ*	1
ΔputA(P*trpEG*-*lacZ*)	Mutant ΔputA harboring the reporter construct P*trpEG*-*lacZ*	This study
GMI1000(P*putA*-*lacZ*)	GMI1000 harboring the reporter construct P*putA*-*lacZ*	This study
ΔputA(P*putA*-*lacZ*)	Mutant ΔputA harboring the reporter construct P*putA*-*lacZ*	This study
GMI1000(P*RSp0162*-*lacZ*)	GMI1000 harboring the reporter construct P*RSp0162*-*lacZ*	2
ΔputA(P*RSp0162*-*lacZ*)	Mutant ΔputA harboring the reporter construct P*RSp0162*-*lacZ*	This study
E. coli		

DH5α	*supE44 lacU169 (80lacZ M15) hsdR17 recA1 endA1 gyrA96 thi-1 relA1 pir*	Laboratory collection
BL21	F-*ompT hsdS (rB-mB-) dcm*+ Tetr *gal* (DE3) *endA*	Laboratory collection
**Plasmid**		
pBT20	Tn5 transposon, Gm^{r}	Laboratory collection
pDBHT2	Expression vector, Kan^{r}	Laboratory collection
pDBHT2-*putA*	pDBHT2 containing *putA*	This study
pDBHT2-*putA*^{K9A}	pDBHT2 containing *putA*^{K9A}	This study
pK18	pK18, sacB^{+}; gene replacement vector; Kan^{r}	Laboratory collection
pK18-*putA*	pK18 containing fragments flanking *putA*	This study
pME2-*lacZ*	Broad-host-range cloning vector; Tet^{r}	Laboratory collection
P*epsA-lacZ*	pME2-*lacZ* containing the promoter of *epsA*	This study
P*putA-lacZ*	pME2-*lacZ* containing the promoter of *putA*	This study
P*RSp0162-lacZ*	pME2-*lacZ* containing the promoter of *RSp0162*	This study
P*trpEG-lacZ*	pME2-*lacZ* containing the promoter of *trpEG*	1
P*phcB-lacZ*	pME2-*lacZ* containing the promoter of *phcB*	This study
P*solI-lacZ*	pME2-*lacZ* containing the promoter of *solI*	This study

[a] Trim^{r}, Kan^{r}, Tet^{r}, Amp^{r}, Gm^{r} and Cat^{r} indicate resistance to trimethoprim, kanamycin, tetracycline, ampicillin, gentamicin and chloramphenicol, respectively.

Supplementary References

1. Song, S. et al. Anthranilic acid from *Ralstonia solanacearum* plays dual roles in intraspecies signalling and inter-kingdom communication. *ISME J* **14**, 2248-2260 (2020).
2. Shen, F. et al. *Ralstonia solanacearum* promotes pathogenicity by utilizing l-glutamic acid from host plants. *Mol Plant Pathol* **21**, 1099-1110 (2020).

Supplementary Table 5 PCR primers used in this study

Primer	Sequence (5’-3’)
For deletion	
*putA*L-F	tgacatgattacgaattcCACGATTACGCCGGCTTCG
*putA*L-R	CGCCCCGTCAGCCGATGGCCATGAGGGTTCCTTTGCTG
*putA*R-F	CAGCAAAGGAACCCTCATGGCCATCGGCTGACGGGGCG
*putA*R-R	gtcgactctagaggatccCTTGTAGCCCAGCTTCTTG
For *in trans* expression	
putA-F	acgaattcccggggatccATGGCTACGACCACCCTCGGGGTCAAG
putA ^{K9A}-F	acgaattcccggggatccATGGCTACGACCACCCTCGGGGTCGCA
putA-R	acggccagtgccaagcttTCAGCCGATGGTCATCAGGCTG
For protein expression	
putA-His-F	catatgggatccgaattcATGGCTACGACCACCCTCGGGGTCAAG
putA-His-R	gagtgcggccgcaagcttTCAGCCGATGGTCATCAGGCTG
For EMSA	
EMSA-*epsA*F	CACGGGCTGCAAGCTCCTG
EMSA-*epsA*R	CGACACGACTGCTTTTCG
EMSA-*epsA*LF	CACGGGCTGCAAGCTCCTG
EMSA-*epsA*LR	CAGGCGGCTTTATGCTGATACGTTTTACAGTTG
EMSA-*epsA*RF	CAACTGTAAAACGTATCAGCATAAAGCCGCCTG
EMSA-*epsA*RR	CGACACGACTGCTTTTCG
EMSA-*phcB*F	GACGCCCTTGACCACGCT
EMSA-*phcB*R	TGCCGGATCGATTTGGTTG
EMSA-*solI*F	TGATGCTTCGATGGCGAC
EMSA-*solI*R	GCCGCCATGAATGAATGTCT
EMSA-*putA*F	CAACTGCTGCTCGAAAGCG
EMSA-*putA*R	CGAGGGTGGTCGTAGCCATG
EMSA-*trpEG*F	AACAAGCCGCACGATTTCAC
EMSA-*trpEG*R	GATGAAGGAATAGCGCCCGA
EMSA-*phcB*1F	TGCGGCCCGCCGAGCGCGT
EMSA- *solI1*F	ATCGGGGGATGATGCTTCGATGGCG
EMSA-*solI1*R	GAAGCATCATCCCCCGATCGATACGG
EMSA-*trpEG1*R	CCACGGCAAGCGCGGTTGCG
For beta-gal	

pME2-l*acZ*-P*putA*-F	CCCAAGCTTCAACTGCTGCTCGAAAGCG
pME2-*lacZ*-P*putA*-R	CCGCTCGAGCGAGGGTGGTCGTAGCCATG
pME2-*lacZ*-P*phcB*-F	CCCAAGCTTGACGCCCTTGACCACGCT
pME2-*lacZ*-P*phcB*-R	CCGGAATTCTGCCGGATCGATTTGGTTG
pME2-*lacZ*-P*solI*-F	CCCAAGCTTTGATGCTTCGATGGCGAC
pME2-*lacZ*-P*solI*-R	CCGGAATTCGCCGCCATGAATGAATGTCT
pME2-*lacZ*-P*RSp0162*-F	CCCAAGCTTGCTCACCGAGACGATGG
pME2-*lacZ*-P*RSp0162*-R	CCGCTCGAGAACCAGCGGCATGCATC
For qRT-PCR	
phcB-F	TATCGCACCTACACCAAGCC
phcB-R	CGCCGAGATAGTTGACCAGG
solI-F	CGGCCCTATCTGCTCAAGG
solI-R	AAGGTCACGCCGATCAGTC
trpE-F	TCGTCTATGCCGATCCGTCCAAG
trpE-R	CGATCTGCATGTCGCCGAAGTTGT
trpG-F	AACCCGGAGCGCATCTGCCTCT
trpG-R	TGTTCTCGATGGTGCTGACCTTGC
RSp0162-F	TACCGACACCACGACCCTGAAGACG
RSp0162-R	CGGCGGACGGATAGATGTAGTTGC
G16S-F	AGGCCTTCGGGTTGTAAAG
G16S-R	CGTAGTTAGCCGGTCCTTATTC
