# Supplementary material for: Proline utilization A controls bacterial pathogenicity by sensing its substrate and cofactors
Source: Commun Biol. 2022 May 25;5:496. doi: 10.1038/s42003-022-03451-4 (PMC9132996; doi:10.1038/s42003-022-03451-4)
Supplement: Supplementary file 3 — Description of Additional Supplementary Files [file 42003_2022_3451_MOESM3_ESM.pdf]

Description of Additional Supplementary Files

File name: Supplementary Data 1
Description: The source data behind the graphs in the paper.

File name: Supplementary Data 2
Description: The raw figures of this article.
