# Supplementary figures and images for: Proline utilization A controls bacterial pathogenicity by sensing its substrate and cofactors
Source: Commun Biol. 2022 May 25;5:496. doi: 10.1038/s42003-022-03451-4 (PMC9132996; doi:10.1038/s42003-022-03451-4)

**Fig.2a**

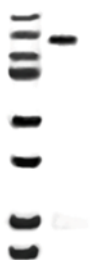

**Fig.2b**

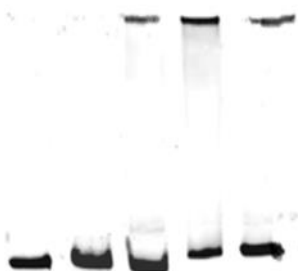

**Fig.3c**

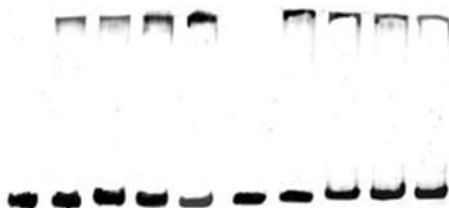

**Fig.5a**

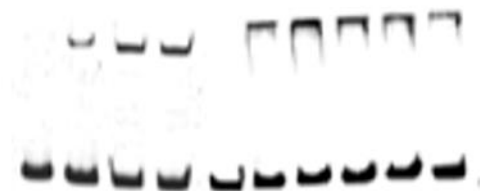

**Fig.5b**

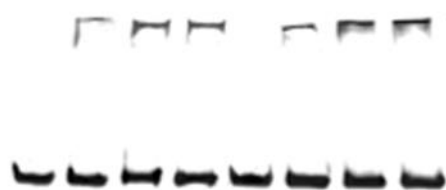

**Fig.5c**

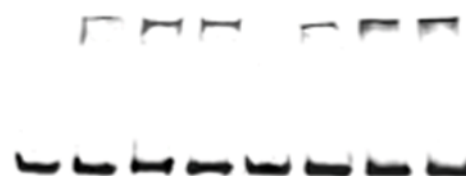

**Fig.S5b1**

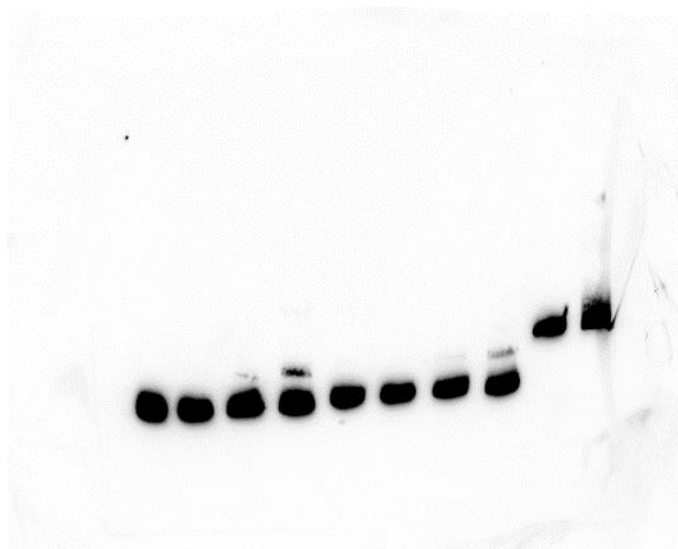

**Fig.S5b2**

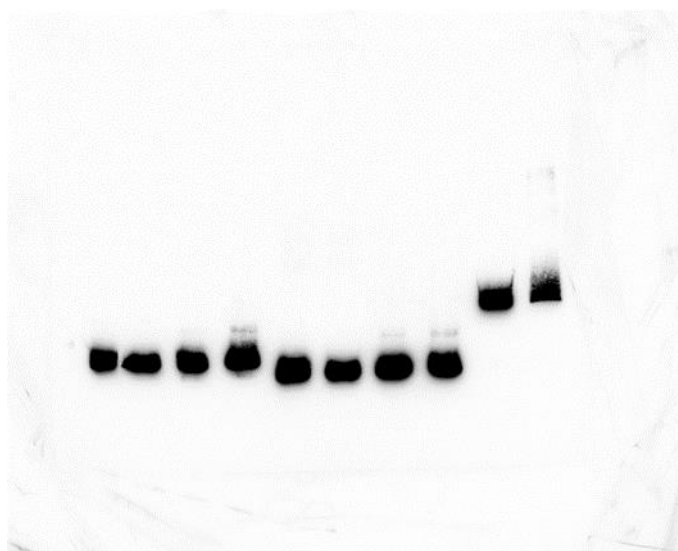

**Fig.S7c**

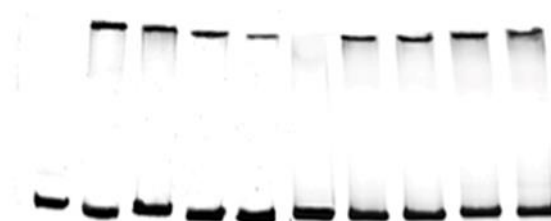

**Fig.S8c**

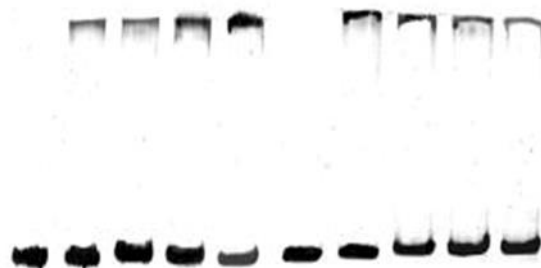

**Fig.S10c**

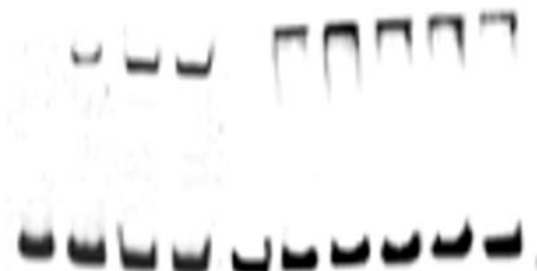

**Fig.S11c**

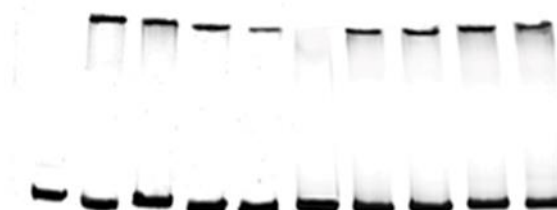

**Fig.S15a&b**

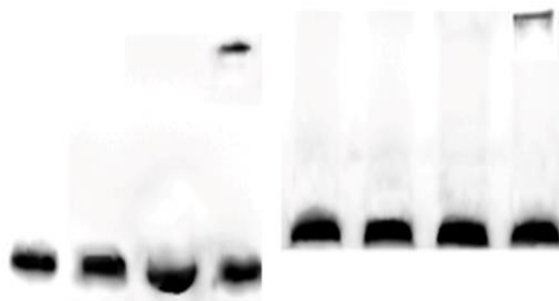

**Fig.S15c**

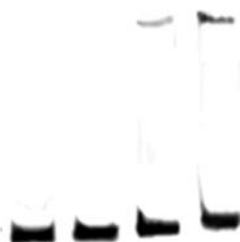

**Fig.S17c**

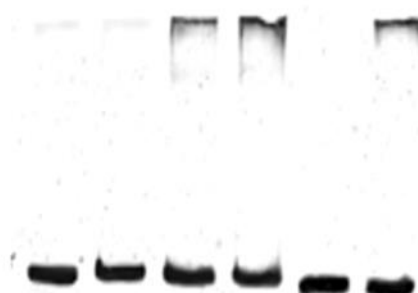

Supplement: Supplementary file 5 — Supplementary Data 2 [file 42003_2022_3451_MOESM5_ESM.pdf]
